# Probing the Effect of Linear and Crosslinked POE-*g*-GMA on the Properties of Asphalt

**DOI:** 10.3390/ma16196564

**Published:** 2023-10-05

**Authors:** Yujuan Zhang, Pei Qian, Peng Xiao, Aihong Kang, Chenguang Jiang, Changjiang Kou, Zhifeng Wang, Yuqing Li

**Affiliations:** 1 College of Civil Science and Engineering, Yangzhou University, Yangzhou 225127, China; 008211@yzu.edu.cn (Y.Z.); mz120210993@stu.yzu.edu.cn (P.Q.); ahkang@yzu.edu.cn (A.K.); changjiang.kou@yzu.edu.cn (C.K.); 2 Research Center for Basalt Fiber Composite Construction Materials, Yangzhou 225127, China; 3 College of Chemistry and Chemical Engineering, Yangzhou University, Yangzhou 225002, China; dx120200067@yzu.edu.cn; 4 Testing Center, Yangzhou University, Yangzhou 225002, China; zfwang@yzu.edu.cn (Z.W.); liyuqing@yzu.edu.cn (Y.L.)

**Keywords:** crosslinking modification of POE-*g*-GMA, asphalt, storage stability, physical properties, thermal oxidation aging resistance

## Abstract

The copolymer ethylene–octene (POE) has good aging resistance and is an inexpensive asphalt additive compared to the styrene–butadiene–styrene copolymer (SBS). However, POE is easy to segregate in asphalt during storage at high temperatures. Grafting glycidyl methacrylate (GMA) onto the molecular backbone of POE (i.e., POE-*g*-GMA) may solve this problem, for the epoxy groups in GMA can react with the active groups in asphalt. Asphalt modified with linear and crosslinked POE-*g*-GMA were prepared, and the hot storage stability, physical properties and thermal oxidation aging properties were discussed in detail. The results show that linear and low-degree crosslinked POE-*g*-GMA-modified asphalts are storage-stable at high temperatures via measurements of the difference in softening points and small-angle X-ray scattering (SAXS) characterizations from macro and micro perspectives. The difference in softening points (ΔSP) between the upper and lower ends is no more than 3.5 °C for modified asphalts after 48 h of being in an oven at 163 °C. More importantly, the crosslinking modification of POE-*g*-GMA can further increase the softening point and reduce the penetration as well as rheological properties via conventional physical property, dynamic shear rheometer (DSR) and multiple-stress creep recovery (MSCR) tests. Furthermore, asphalt modified with crosslinked POE-*g*-GMA reveals better aging resistance via measurements of the performance retention rate and electron paramagnetic resonance (EPR) characterizations after a rolling thin film oven test (RTFOT). This work may provide further guidelines for the application of polymers in asphalt.

## 1. Introduction

**Figure 1:**
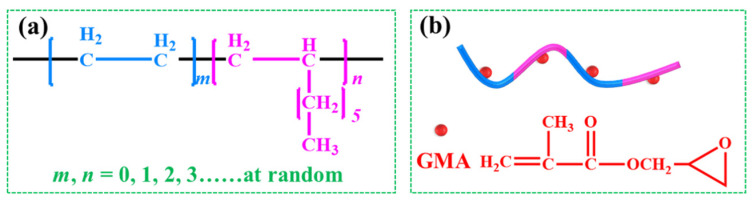
(**a**) The chemical formula of POE and (**b**) structural model of POE-*g*-GMA.

Asphalt modified with polymers has a long history because polymers can improve the physical and rheological performance of asphalt. The triblock copolymer styrene–butadiene–styrene (SBS) can remarkably enhance the high-temperature rutting resistance and low-temperature cracking resistance of a matrix asphalt simultaneously, and is the most widely used modifier for asphalt [1]. However, SBS-modified asphalt faces several challenges, including poor aging resistance caused by the presence of double bonds in the backbone and high costs [2,3]. The copolymer ethylene–octene (POE) is one kind of polyolefin elastomer, and its phase structure is similar to that of SBS [4]. According to the chemical formula (Figure 1a), as a kind of thermoplastic elastomer with a saturated main chain, POE has excellent heat resistance and oxygen aging performance [5]. Moreover, it is much cheaper than SBS is. Therefore, asphalt modified with POE is necessary to be regarded as the research object.

It is difficult for polymer-modified asphalt to achieve compatibility and it easily tends toward phase separation, which mainly arises from differences in molecular structure and weight, density and viscosity [6]. In view of the kinetic point, the system of polymer-modified asphalt (PMA) tends to segregate at high temperatures [7], which restricts the application of PMA in large-scale settings. In general, it contains a high probability to form a homogenous mixture between materials with similar polarities after physical blending [8]. However, the polarity of POE is weak, resulting in the poor compatibility of the two phases and low thermal storage stability. Therefore, it is difficult to prepare POE-modified asphalt with good compatibility only via mechanical blending [9].

Many efforts have been devoted to improving the compatibility between the polymer and asphalts phase, and chemical modification is certified as an effective approach via a chemical reaction of both phases [10]. It is known that some functional groups (e.g., epoxy groups) can react with the several active groups (e.g., hydroxyl groups) that exist in asphalt. Therefore, introducing functional groups into the molecular chain of a polymer, specifically a functional polymer, is one of the most popular methods to alleviate and overcome the problem of the poor compatibility of PMA [6]. Thermoplastic elastomers grafted with maleic anhydride (MAH) or glycidyl methacrylate (GMA) and the copolymers of ethylene-containing epoxy groups, which can be referred to as functionalized polymers, have been widely studied as asphalt modifiers [11,12]. Therefore, grafting GMA onto the molecular backbone of POE (Figure 1b) may solve the problem of POE segregation in asphalt. The production technology of POE grafted using GMA (POE-*g*-GMA) has become more and more advanced. Moreover, the crosslinking modification of a polymer can further improve its mechanical properties, thermal stability, and aging resistance, and the designability of the crosslinked network structure leads to the adjustability of material properties.

In this work, different crosslinked POE-*g*-GMAs were synthesized by changing the content of dicumyl peroxide (DCP) using the melt blending strategy, and then asphalt was modified via linear and crosslinked POE-*g*-GMA, respectively. The storage stability, physical and rheological properties were evaluated, and the resistance of thermal oxidation aging was also discussed in detail. POE-*g*-GMA improves the properties of asphalt, and compared with that modified with POE, POE-*g*-GMA-modified asphalt shows superior storage stability. Other than that, low-degree crosslinked POE-*g*-GMA has a small effect on storage stability, and can further enhance the properties of asphalt, which may satisfy the multi-functional needs of modern transportation.

## 2. Materials and Methods

### 2.1. Materials

Penetration 70 asphalt was used as the research object in this work, and the physical properties are listed in Table 1. POE and POE-*g*-GMA were provided by Xiamen Coace Chemical Co., Ltd. (Xiamen, Fujian, China). The melting index of POE-*g*-GMA is 6.0 g/min (load of 2.16 kg, at 190 ℃), and the grafted rate is 1.2–2.0%. Dicumyl peroxide (DCP) was obtained from Sinopharm Chemical Reagent Co., Ltd. (Shanghai, China).

### 2.2. Preparation Methods

#### 2.2.1. Preparation of Crosslinked POE-*g*-GMA

Before processing, POE-*g*-GMA was dried at 80 °C for 6 h in a vacuum-dried oven. A Haake internal mixer (Thermo Scientific Co., Waltham, MA, USA) was employed to prepare crosslinked POE-*g*-GMA via melt blending with different DCP contents at 190 °C and 40 rpm for 5 min. The as-prepared samples were cut into small pieces with scissors. Hereafter, the crosslinked POE-*g*-GMA samples are marked as PG-*x*, where *x* is the concentration (wt%) of DCP in blends.

#### 2.2.2. Preparation of the Modified Asphalts

First, matrix asphalt was melted at 170 °C until it could completely flow in the container, and different modifiers with predetermined ratios were added into the flowing asphalt, which was left to swell at 170 °C for 30 min after operating manual stirring. Then, the asphalt blends were sheared at about 170 °C for 30 min with a speed of 5000–6000 rpm using a high-speed shear mixer. At last, the modified asphalt was incubated at 170 °C for 30 min. The asphalt modified with y wt% POE-*g*-GMA is coded as A-*y*PG, while the asphalt modified with PG-*x* is referred to as A-3PG-*x*. The content of PG-*x* was fixed at 3 wt%. A detailed composition of the blends is shown in Table 2. As a comparison, asphalt modified with 3 wt%POE (A-3P) was chosen as the control sample.

#### 2.2.3. Laboratory Aging

A rolling thin film oven test (RTFOT) was carried out to simulate the short-term thermal oxygen aging that happens to different asphalts (for 85 min, at 163 °C with rotation and the blowing of air at 4 L/min) according to ASTM D2872.

### 2.3. Characterizations

#### 2.3.1. Attenuated Total Reflectance Fourier Transform Infrared Spectroscopy (ATR-FTIR)

The Cary 610/670 micro-infrared spectrometer produced by Varian Company (Palo Alto, CA, USA) was employed to carry out ATR-FTIR for analyzing changes in chemical structure. The scanning range of the instrument is 4000–500 cm^−1^ with a resolution of 4 cm^−1^, and it has an accumulation of 32 scans continuously.

#### 2.3.2. Gel Contents Measurement

The gel content analysis of crosslinked POE-*g*-GMA was carried out via extraction with toluene in a Soxhlet apparatus at 130 °C for about 72 h, which can be used to qualitatively analyze the degree of crosslinking of POE-*g*-GMA. The insoluble products obtained via filtration were dried at 130 °C in an oven to a constant weight. The gel contents (%) were measured using Equation (1):

(1) $$\mathrm{Gel}\;\mathrm{content}\;=w_{2}/w_{1}\times 100\%$$

where *w*_1_ is the weight of the as-prepared samples without extraction, while *w*_2_ is the weight of the insoluble products.

#### 2.3.3. Differential Scanning Calorimetry (DSC)

A DSC (8500, PerkinElmer Co., Wilmington, DE, USA) test was performed to detect the melting behaviors of PG-*x* (~10 mg). The heat program was as follows: heating from −65 to 100 °C with a heating rate of 5 K/min. The measurements were conducted in a nitrogen atmosphere.

#### 2.3.4. Storage Stability and Conventional Physical Properties

The test on storage stability under high- temperatures was carried out in accordance with the standard T 0661-2011 [13], while the ΔSP between the top and bottom of the aluminum tube was used to evaluate the storage stability of modified asphalts at high temperatures. Modified asphalt with a ΔSP of less than 3.5 °C can be supposed to have good storage stability. The tests on conventional physical properties, including penetration, softening point and ductility, were carried out in accordance with standards T 0604-2011, T 0606-2011 and T 0605-2011, respectively [13]. Three replications of each test were performed to obtained averages for each test project.

#### 2.3.5. Small-Angle X-ray Scattering (SAXS)

The compatibility of modified asphalts in view of microscopic perspective was characterized using a Nano STAR small-angle X-ray scatter (SAXS) meter produced by Bruker, Saarbrucken, SL, Germany. The asphalt was wrapped in tinfoil and pressed into sheets of a thickness of less than 1 mm. The test was performed at room temperature, and the incident X-rays of CuKα radiation (1.54 A) were monochromated using a cross-coupled Göbel mirror and passed through the sheet sample. The distance between the sample and detector was calibrated using silver behenate, giving a scattering vector q range of 0.07 to 0.25 nm^−1^.

#### 2.3.6. Dynamic Shear Rheometer (DSR)

*Temperature Sweep Test*

A DHR-2 rotational rheometer equipped with a pair of 25 mm parallel plates was employed to conduct the rheological experiments, and was produced by TA Co., New Castle, DE, USA. The measurements were conducted at 10 rad/s with a 1.0 mm gap and 0.2% strain, and the range of the temperature sweep test was from 40 to 80 °C.

*Multiple-Stress Creep and Recovery Test*

The MSCR test was conducted at 64 °C to evaluate the resistance to permanent deformation of different asphalts under shear stress values of 0.1 kPa and 3.2 kPa.

#### 2.3.7. Morphological Characterization

A LSM700-3D laser microscope (CARL ZEISS, Co., Oberkochen, Germany), which was equipped with a blue filter system with a wavelength of excitation ranging from 390 to 490 nm, was employed to investigate the morphology of the modified asphalts at a magnification of 200. The heated, liquid modified asphalts were poured into a square mold, and then left to cool down to room temperature to obtain a flat surface.

#### 2.3.8. Electron Paramagnetic Resonance (EPR) Test

The free radicals produced due to aging were detected via A300 EPR spectroscopy (Bruker, Saarbrucken, Germany). The test conditions were as follows: the sweep time was 167.772 s, the time constant was 163.840 ms, and the sweep width was 500 G. After the RTOFT test, the sample was dropped into trichloroethylene immediately to stop free radicals from quickly quenching. The solution was extracted to be measured with a capillary, and then the capillaries containing the samples were placed in a standard 4 mm quartz sample tube.

## 3. Results and Discussion

### 3.1. Formation of Crosslinked POE-g-GMA

**Figure 2:**
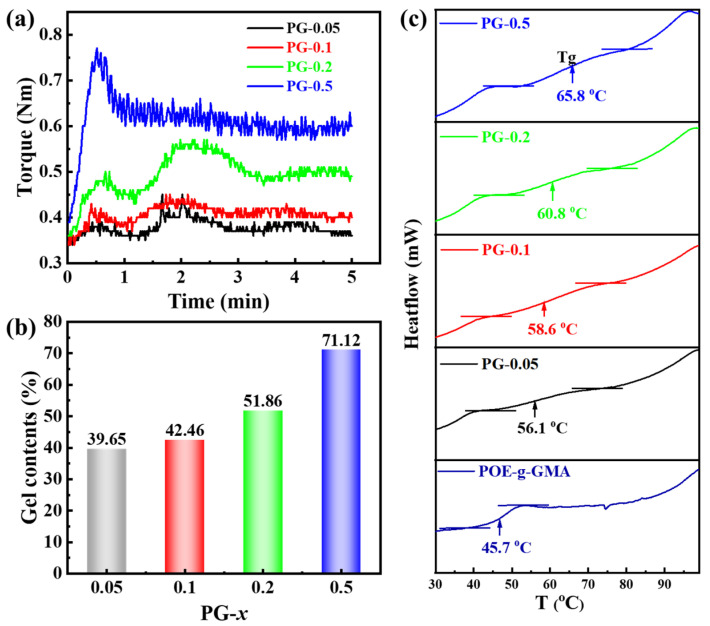
(**a**) Torque evolution curves, (**b**) gel contents and (**c**) DSC heating curves of PG-*x*.

The crosslinking modification of POE-*g*-GMA was performed via the melt blending of POE-*g*-GMA with DCP at 190 °C. Figure 2a gives the torque curves of crosslinked POE-*g*-GMA with different contents of DCP. As the mixing time increased, the torque gradually increased at the beginning of mixing, which indicates the occurrence of a crosslinking reaction. The torque also increased with the increasing content of DCP, indicating that the density and degree of crosslinking increase with the loading of DCP [14]. The gel contents of the PG-*x* were determined by dissolving it in toluene and via weighting. Figure 2b shows that with the increased loading of DCP from 0.05 to 0.5%, the gel contents of PG-*x* increased. The gel content increased from 39.65% to 71.12% while the content of DCP was 0.05 wt% and 0.5 wt%, respectively. The glass transition temperature (Tg) of PG-*x*s was determined via a DSC trace. As shown in Figure 2c, the Tg gradually shifts to a higher temperature with the growth content of DCP. The Tg of PG-0.5 is 65.8 °C, which compared to that of POE-*g*-GMA represents an increase of about 10 °C. The growth of Tg indicates the restriction of the chain mobility of the polymer [15]. The results indicate that it is possible to successfully conduct the crosslinking modification of POE-*g*-GMA with DCP, and that it is easy to adjust the degree of crosslinking by varying the content of DCP.

**Figure 3:**
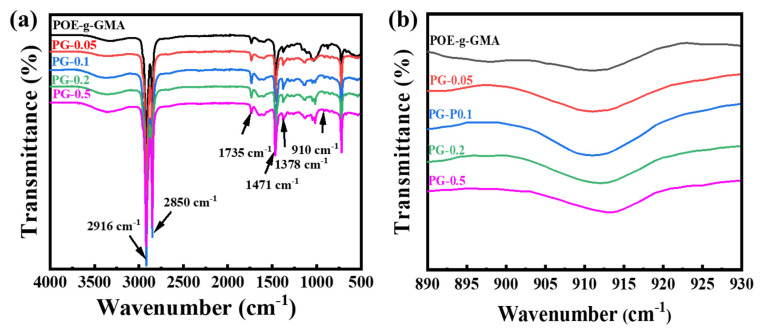
(**a**) The range of 500–4000 cm^−1^ and (**b**) 890–930 cm^−1^ in the FT-IR spectra of POE-*g*-GMA with various DCP contents.

The characterization of FT-IR is displayed in Figure 3. As shown in Figure 3a, the crosslinking modification of POE-*g*-GMA has a small effect on the functional groups. A peak around 1735 cm^−1^ refers to the stretching vibration of C=O in GMA. The peak located at about 2916 cm^−1^ and 2850 cm^−1^ refers to stretching vibration of C-H_2_, and the peak located at 1471 cm^−1^ and 1378 cm^−1^ refers to the bending vibration of C-H and C-H_3_. The epoxy group located at about 910 cm^−1^ is the active group of glycidyl methacrylate (GMA), which can react with the carboxyl, carbonyl and other active groups in the matrix asphalt to enhance high-temperature stability. As shown in Figure 3b, the characteristic peak of the epoxy groups still existed in the crosslinked POE-*g*-GMA with various DCP contents. The results demonstrate that the olefin chain of POE-*g*-GMA was attacked by the free radicals generated by DCP without the consumption of the epoxy groups during crosslinking modification. In the other words, the crosslinked POE-*g*-GMA prepared in this paper still exhibited reactivity, and could further react with the matrix asphalt in the following melt blending procedure.

### 3.2. Storage Stability of Modified Asphalts

**Figure 4:**
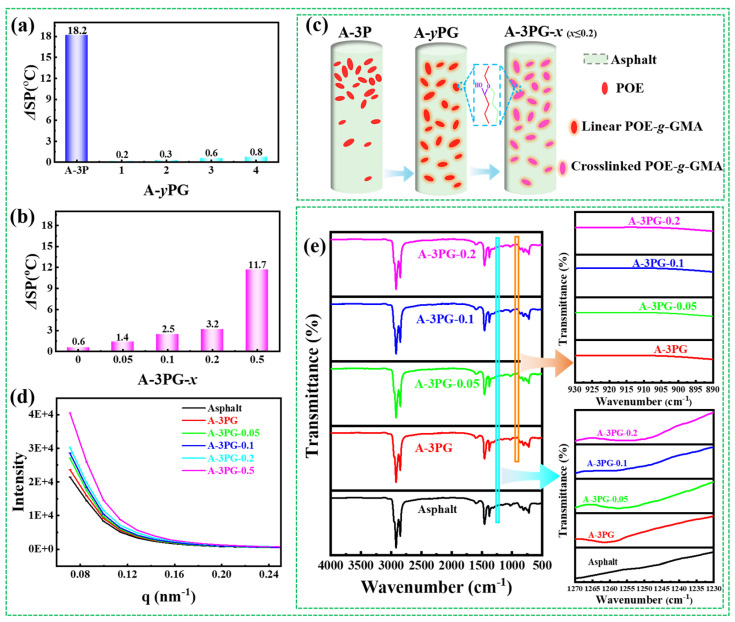
The storage stability of (**a**) A-*y*PG and (**b**) A-3PG-*x*; (**c**) the schematics for storage stability, (**d**) the SAXS spectra and (**e**) the FT-IR spectra of different modified asphalts.

The storage stability of modified asphalts at high temperatures is commonly evaluated from a macro perspective via a calculation of the value of the ΔSP, and the smaller the ΔSP value, the more stable it is. According to the literature, asphalt modified with polyolefin usually has low high-temperature storage stability [16,17,18], which has serious adverse impacts on pavement performance. Values of the ΔSP for different modified asphalts and microstructure characteristics are studied in this paper. As shown in Figure 4a, the ΔSP value of A-3P is 18.2 °C, indicating obvious phase separation between POE and asphalt in the process of hot storage. However, POE-*g*-GMA is stably and uniformly dispersed in asphalt, which is confirmed by the value of the ΔSP (Figure 4a). Although the ΔSP value of modified asphalt slightly increases with the rise in the POE-*g*-GMA content, all values are less than 1.0 °C, which is indicative of satisfactory compatibility. This may be a result of the reaction that happens between the epoxy groups in POE-*g*-GMA and the hydroxyl, carboxyl, and other active groups in the asphalt during processing, which improved its storage stability [6,19,20]. The results of the ΔSP of the asphalt modified with crosslinked POE-*g*-GMA are given in Figure 4b. With the rise in *x*, the value of the ΔSP increases, and asphalt modified with weakly crosslinked POE-*g*-GMA (*x* ≤ 0.2) shows good storage stability with a ΔSP value of less than 3.5 °C, while the ΔSP of A-3PG-0.5 is more than 3.5 °C, reaching 11.7 °C. The results indicate that the crosslinking modification of POE-*g*-GMA has an adverse impact on the compatibility of the modified asphalts. This is because the migration of molecular chains is restricted by the crosslinking network structure, resulting in a decrease in the reactivity of epoxy groups in PG-*x* [14]. Hence, the thermal storage stability of asphalt modified with highly crosslinking PG-*x* is poor. Figure 4c describes the schematics of the modification and storage stability of different modified asphalts.

Figure 4d displays the SAXS spectra of different asphalts. It can be seen that all samples have a high scattering intensity, and that the scattering intensity of A-3PG-*x* increases with the increase in *x*, which is especially the case for A-3PG-0.5. The high scattering intensity in the SAXS spectra arises from the fluctuations in molecular density caused by phase separation [21]; thus, the results indicate that the composition of asphalt itself is very complex, and the crosslinking modification of POE-*g*-GMA exacerbates the phase separation of modified asphalt at a micro level. The result is consistent with that of the macroscopic compatibility test.

Figure 4e depicts the FT-IR spectra of different modified asphalts. The results show the disappearance of the epoxy group (~910 cm^−1^) and the emergence of new characteristic peaks (~1255 cm^−1^), which may be attributed to C-O in the aromatic ether. FT-IR spectra confirm the existence of the reaction between POE-*g*-GMA and asphalt. Therefore, asphalt modified with POE-*g*-GMA shows good storage stability.

### 3.3. Penetration, Softening Point and Ductility

The test of penetration was conducted to evaluate the consistency and hardness of modified asphalt, and the results of penetration are given in Table 3. With the growth content of POE-*g*-GMA, the penetration of modified asphalts gradually decreases, and the penetration of asphalt decreases from 63.2 to 40.2 after loading with 4 wt% POE-*g*-GMA, which indicates that the hardness and consistency of asphalt can be enhanced via modification with POE-*g*-GMA. The penetration of asphalt modified with PG-*x* slightly decreases, indicating that weakly crosslinked POE-*g*-GMA has a small impact on the consistency and hardness of asphalt [22]. The softening point was determined to evaluate the properties under high temperatures, and properties under low temperatures were evaluated via the ductility under 10 °C. All the test data are given in Table 3, and the table shows that along with the rise in the loading of POE-*g*-GMA, the softening point gradually increases. The softening point of asphalt increases from 47.7 °C to 54.9 °C after being modified with 4 wt% POE-*g*-GMA. The softening point of A-3PG-*x* continuously increases with the increase in *x*. When *x* = 0.2, the softening point continues to increase from 52.6 °C to 59.2 °C, indicating that the weak-crosslinking modification of POE-*g*-GMA can further enhance the properties under high temperatures. A ductility test was performed at 10 °C. The results in Table 3 show that ductility reduces from 62.0 cm to 15.5 cm after modification with 1 wt% POE-*g*-GMA, reflecting that POE-*g*-GMA has a visible harmful influence on ductility. However, ductility slightly increases as the content of POE-*g*-GMA continues to increase. The ductility of asphalt modified with PG-*x* decreases with an increase in *x*, indicating that crosslinking modification is not conducive to its properties under low temperatures.

### 3.4. Dynamic Rheological Properties

**Figure 5:**
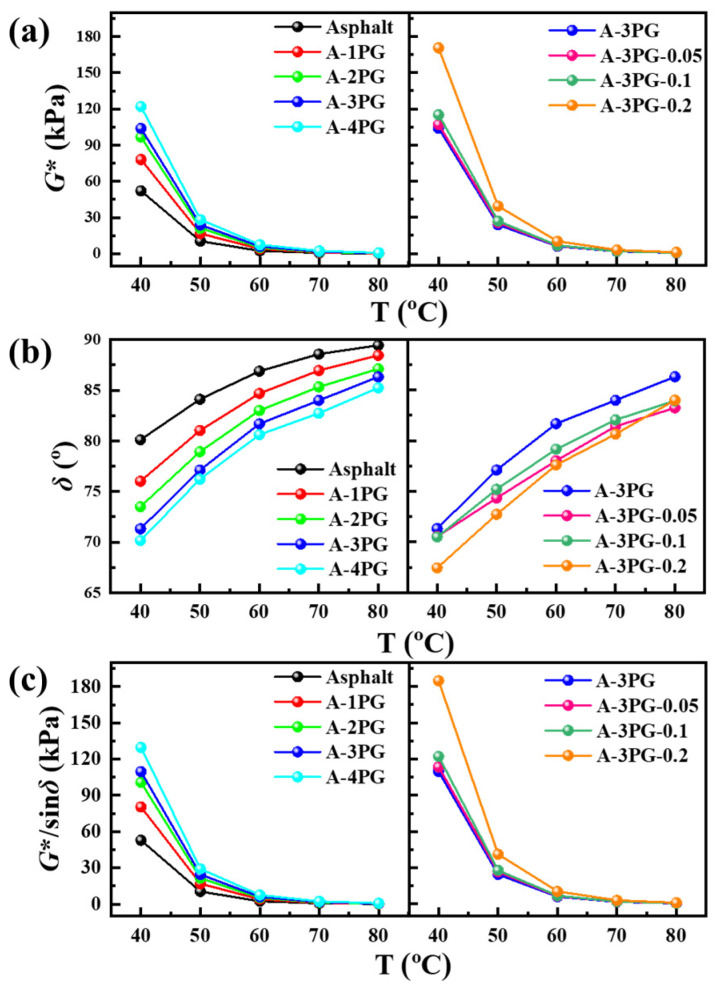
(**a**) The complex modulus, (**b**) the phase angle and (**c**) the rutting factor of different modified asphalts.

The difference in chemical composition or structure between modifier and asphalt has an impact on its rheological performance, which is strongly associated with the processing and paving of the asphalt mixture and the properties of the pavement [23,24]. Therefore, it is important to study the rheological performance of modified asphalts, including their complex shear modulus (G*), phase angle (*δ*) and rutting factor (G*/sin*δ*). G* and *δ* were directly obtained via a DSR test. G* represents the stiffness and the ability of resistance to shear deformation. δ is the ratio of the elastic to the viscous component of asphalt, and a smaller δ indicates that there are more elastic components in the asphalt, reflecting that it is easier to be deformed and that there are more unrecoverable parts in the deformation of asphalt. The ability to resist high-temperature rutting can be evaluated by calculating the value of G*/sin*δ*, which is an important index and defined in the Superpave specification [25]. The G*, *δ* and G*/sin*δ* curves with the temperature for the matrix asphalt and different modified asphalts are shown in Figure 5. As the temperature increases, G* and G*/sin*δ* become smaller and smaller, while *δ* becomes larger and larger.

In Figure 5a, G* increases along with the increasing content of POE-*g*-GMA, and the G* of A-3PG-*x* increases with the rise in *x*, reflecting that the weak crosslinking modification of POE-*g*-GMA further enhances the stiffness and resistance to shear deformation of asphalt [26]. The δ of the modified asphalts is smaller than that of the matrix asphalt (Figure 5b), and the change rule is opposite to that of G*, which is indicative of stronger elastic properties [27]. The rutting factor is displayed in Figure 5c, and the change rule is consistent with that of G*. The results of G*/sin*δ* show that the anti-rutting factor of asphalt is improved via modification with linear POE-*g*-GMA, and crosslinked POE-*g*-GMA can further strengthen the rutting resistance of modified asphalts [28]. Overall, it is beneficial to the improvement of the properties of asphalt under high temperatures via modification with linear and crosslinked POE-*g*-GMA.

**Figure 6:**
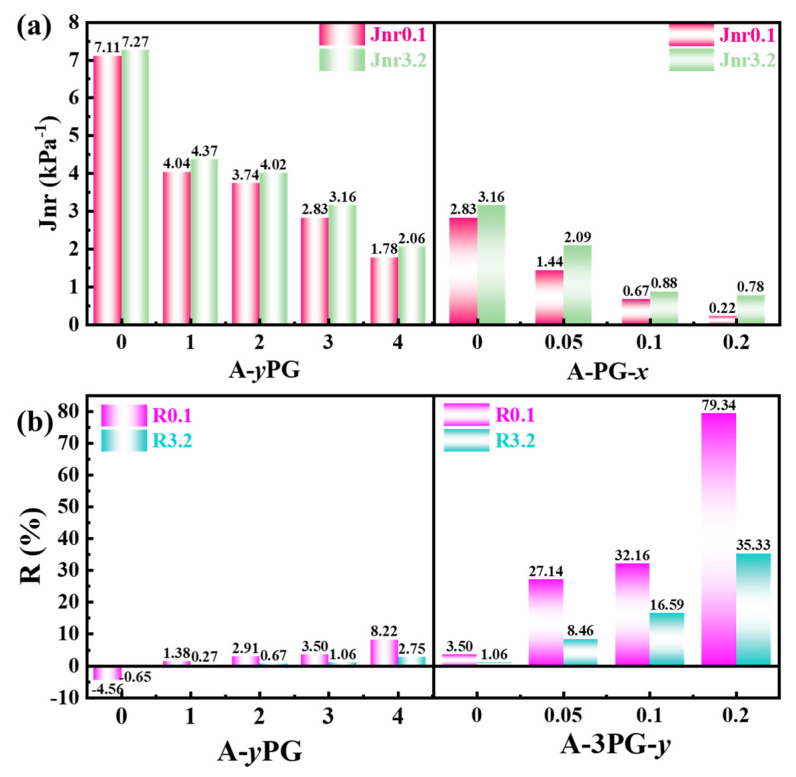
(**a**) Non-recoverable creep compliance and (**b**) Percent recovery of different modified asphalts.

The evaluation parameters, non-recoverable creep compliance (Jnr) and percent recovery (R), were calculated and are shown in Figure 6; these parameters are more representative and convincing than are rutting parameter for evaluating the resistance of asphalt to rutting deformation under high temperatures. Figure 6a shows the Jnr of different modified asphalts at 0.1 and 3.2 kPa (Jnr01 and Jnr3.2), while the R values (R01 and R3.2) are given in Figure 6b. Jnr01 and Jnr3.2 of POE-*g*-GMA-modified asphalts are lower than that of unmodified asphalt, and decrease with the increasing content of POE-*g*-GMA, which indicates that POE-*g*-GMA improves rutting resistance. Moreover, crosslinked POE-*g*-GMA can further enhance the high-temperature performance. The Jnr value represents the unrecoverable strain after removing the preset load, while R is employed to characterize the ability of asphalt to restore its original state. As shown in Figure 6b, the R of POE-*g*-GMA-modified asphalts is higher than that of unmodified asphalt, and crosslinked POE-*g*-GMA significantly increases the value of R, especially in the case of A-3PG-0.2. Overall, it is beneficial to the improvement of the rheological properties of asphalt to modify it with linear and crosslinked POE-*g*-GMA. This is because that the functional groups in POE-*g*-GMA have the ability to react with the acidic compound in asphalt and therefore the network structure. The movement of the modified asphalt molecule was restricted by the network structure. As a result, the flow and deformation of asphalt were restricted under high temperatures.

### 3.5. Morphology of Modified Asphalts

**Figure 7:**
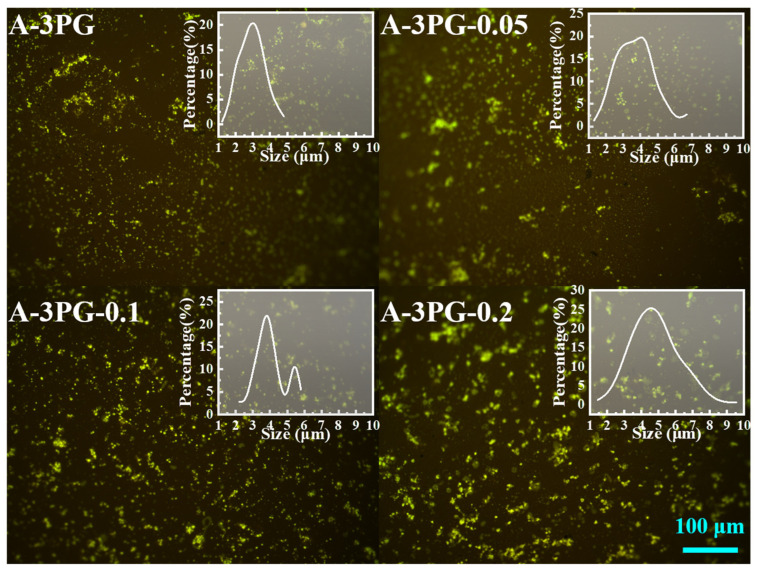
The fluorescence micrographs of A-3PG-*x* before thermal oxidation aging.

The dispersibility of a polymer in asphalt is extremely important when it comes to the properties of asphalt [24,29]. The fluorescence microscope test is considered to be the simplest but most valuable method to analyze the morphology of asphalt modified with a polymer [30,31]. The distribution and phase structure of linear and crosslinked POE-*g*-GMA in the asphalt was characterized using a 3D laser microscope, and the fluorescent images with a 200× magnification are shown in Figure 7. The POE-*g*-GMA particles with different degrees of crosslinking lit up with a greenish-yellow glow; they show a dot structure and good dispersion in the asphalt. As the figure inserted in Figure 7 shows, the grain size distribution curve gradually shifted toward a larger size, indicating that the particle size of PG-*x* increases with the increase in the degree of crosslinking, and the average sizes are 2.95 μm, 3.72 μm, 4.02 μm and 4.85 μm. This may be because crosslinking modification increased the viscosity of the polymer, and made it more and more difficult for the POE-*g*-GMA phase to break into smaller sizes [14]. Weak crosslinking had a small impact on the dispersibility of POE-*g*-GMA.

### 3.6. Thermal Oxidation Aging Resistance of Modified Asphalts

The comparison of physical parameters, including the penetration, softening point, and ductility of asphalts, obtained before and after RTFOT experiment can be applied to evaluate the aging resistance of asphalt, and the results are given in Table 4. It can be seem that the performance change trend of PG-*x* modified-asphalt is free of thermal oxidation aging. Furthermore, the physical parameters of modified asphalt after aging change in the same way as do those of unmodified asphalt; the penetration and ductility of all samples decrease, but the softening point is higher than that before aging.

**Figure 8:**
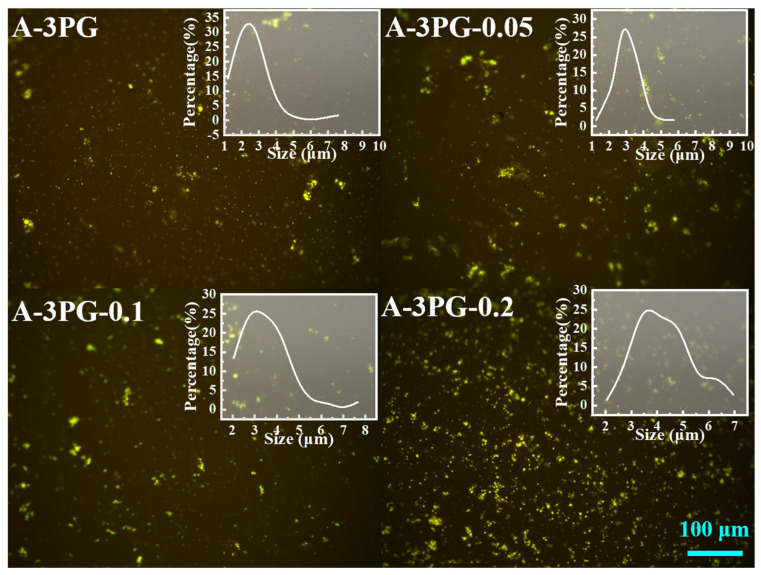
The fluorescence micrographs of A-3PG-*x* after thermal oxidation aging.

Figure 8 displays the change in both the morphology of modified asphalts and the particle size of the polymer after aging. Because of degradation [32], the average size of polymer particles decreases to 2.65 μm, 3.02 μm, 3.49 μm and 4.3 μm. Moreover, the results of fluorescence microscopy show the difference between the residual dosages of PG-*x* in asphalt after aging, and the greater the *x*, the more residual dosages. This is because that crosslinking modification can inhibit the degradation of polymers during aging [33].

**Figure 9:**
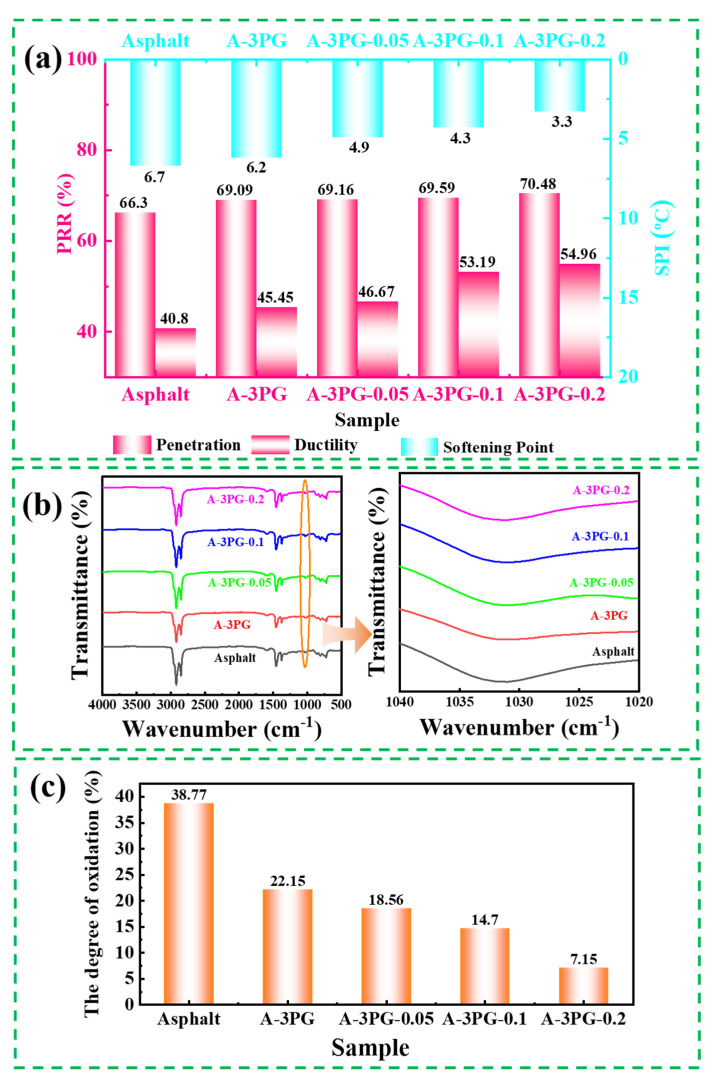
(**a**) The aging indexes of different modified asphalts; (**b**) the FT-IR spectra and (**c**) degree of oxidation of different modified asphalts after thermal oxidation aging.

The property retention ratio (PRR) of penetration and ductility and the softening point increment (SPI) can be defined as aging indexes and they are useful for evaluating the extent of aging in asphalt; a large PRR and small SPI correspond to a low degree of aging [34,35,36]. Both the PRRs and SPI were conducted to study the aging resistance of the matrix and modified asphalts in this paper. The PRR and SPI and were calculated according to Equations (2) and (3), and the results of PRR and SPI are shown in Figure 9a. For all asphalts, the retention ratio of ductility is lower than that of penetration, indicating that aging has a great impact on ductility. The PRRs of all modified asphalts are greater than those of the matrix asphalt, while the SPIs are smaller than those of the matrix asphalt, indicating that both linear and crosslinked POE-*g*-GMA can effectively enhance the thermal oxidation aging resistance of asphalt. Modified asphalts have good anti-aging performance, mainly because POE-*g*-GMA is a kind of polymer with a saturated molecular backbone [37]. It is noteworthy that with an increase in x, the change trends of PRR and SPI are consistent for all asphalts, while PRRs gradually increase and SPIs decrease, reflecting a reduction in the aging degree of asphalt. The crosslinking modification of POE-*g*-GMA can further enhance the aging resistance of modified asphalt.

(2) $$\mathrm{PRR}=\frac{\mathrm{Property}\;\mathrm{after}\;\mathrm{aging}}{\mathrm{Property}\;\mathrm{before}\;\mathrm{aging}}\times 100\%$$

(3) SPI=Softening point after aging−Softening point before aging

ATR-FTIR was employed to detect the change in characteristic functional groups in asphalt during thermal oxidation aging. Oxygen-containing functional groups (e.g., sulfoxide and carbonyl) are often used to reflect the aging characteristics and indicators of asphalt. In this work, the sulfoxide index (*I*_S=O_) was applied to reflect the changes in S=O in the asphalt. The vibration of the sulfoxide S=O functional group is located at about 1031 cm^−1^ [38], and the related FTIR spectra are shown in Figure 9b. The *I*_S=O_ can be determined as follows [39]:

(4) $$I_{S=O}=\frac{A_{1031}}{\sum A}$$

where $A_{1031}$ is the peak area of S=O, and ∑A represents the total peak area sum from 4000 cm^−1^ to 500 cm^−1^. The degree of oxidation (%) was used to study the impact of PG-*x* on the anti-aging performance of asphalt, and was calculated via the following equation:

(5) $$\mathrm{The}\;\mathrm{degree}\;\mathrm{of}\;\mathrm{oxidation}=\frac{I_{S=O}^{*}-I_{S=O}}{I_{S=O}}\times 100\%$$

where $I_{S=O}^{*}$ is the sulfoxide index after aging. As shown in Figure 9c, the result of *I*_S=O_ of the matrix asphalt is much greater than that of modified asphalts, and decreases with the increased degree of the crosslinking of POE-*g*-GMA. This indicates that PG-*x* inhibits the oxidation reaction occurring in asphalt during thermal -oxidation aging. Moreover, the greater the crosslinking of POE-*g*-GMA, the better the effect.

**Figure 10:**
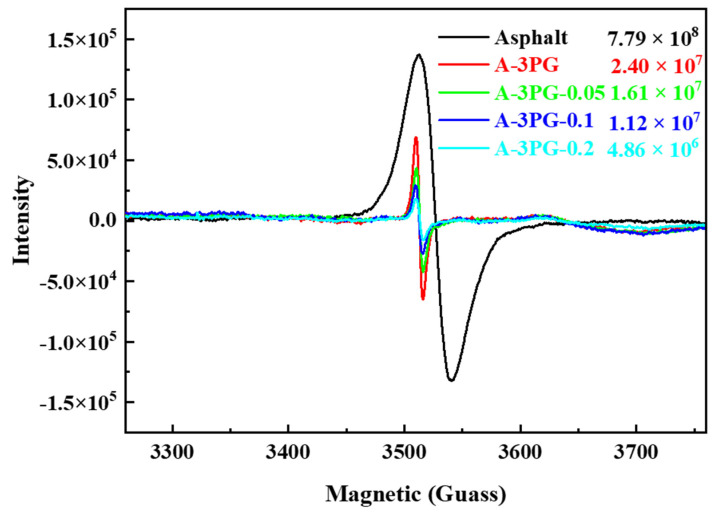
EPR spectra of the different modified asphalts after thermal oxidation aging.

Free radical theory can be applied to explain the mechanism of the thermal oxidation aging of asphalt [40,41]. The EPR technique has provided a unique and powerful tool to elucidate radical mechanisms [42,43]. The free radicals produced via thermal oxidation aging in matrix and modified asphalts were detected via EPR measurements. Via the double integration of experimental data, the overall yield of radicals was semi-quantitatively determined. As shown in Figure 10, the signal of the intensity of asphalt is the highest, while the signal intensity dramatically decreases after modification, and reduces with the increased degree of the crosslinking of POE-*g*-GMA. The results indicate that the yield of radicals decrease. This is because crosslinking places a restriction on the mobility of molecular chain segments, and the movement ability of macromolecular free radicals decreases, slowing down the reaction rate of free radical chain growth [44]. As a result, the yield of radicals decreases, which is indicative of good characteristics of resistance to thermal oxidation aging. Therefore, it is effective to enhance the thermal aging resistance of modified asphalt by regulating the mobility of polymer molecular chains through the crosslinking modification of a polymer modifier.

## 4. Conclusions

POE-*g*-GMA was taken as the research object of this paper, and it was crosslinked via melt blending with DCP. Subsequently, samples of asphalt modified with linear and crosslinked POE-*g*-GMA were prepared. Thereout, the effect of the polymer molecular network structure on the properties of asphalt was studied. The conclusions are as follows:

1. The compatibility between linear or crosslinked POE-*g*-GMA and asphalt can be evaluated from macroscopic and microscopic perspectives via measuring the difference in softening points and SAXS characterizations. It is found that asphalt modified with linear or low-degree-crosslinked POE-*g*-GMA shows excellent hot storage stability compared to POE-modified asphalt. However, high crosslinking may restrain the reactivity of epoxy groups in POE-*g*-GMA, which has an adverse effect on its compatibility with asphalt.
2. With the modification of POE-*g*-GMA, the penetration reduces and the rheological properties increase as well as the softening point, which endows asphalt with a good ability to resist high-temperature rutting, while the crosslinking modification of POE-*g*-GMA further enhances the modification effect.
3. Moreover, the crosslinking modification of POE-*g*-GMA has a positive impact on the thermal oxidation aging resistance of modified asphalt for oxidation reactions are inhibited during the process of aging, and the movement ability of macromolecular free radicals are restricted, thereby slowing down the reaction rate of free radical chain growth. The EPR technique provided a unique and powerful tool to elucidate the radical mechanisms.

Because of its excellent performance and low cost, POE-*g*-GMA may be chosen as a good candidate to modify asphalt with good engineering benefits.

## Figures and Tables

**Table 1:** The basic properties of Pen70 asphalt.

Properties	Value	Standard
Penetration (25 °C, 100 g, 5 s) (0.1 mm)	63.2	ASTM D5
Softening Point (°C)	47.7	ASTM D36
Ductility (5 cm/min, 10 °C) (cm)	62.0	ASTM D113
Viscosity (60 °C), Pa·s	213	ASTM D2171

**Table 2:** Fabrication of asphalts modified with PG-x.

Code	*x*	Content of PG-*x* (wt%)
A-1PG	0	1
A-2PG	0	2
A-3PG	0	3
A-4PG	0	4
A-3PG-0.05	0.05	3
A-3PG-0.1	0.1	3
A-3PG-0.2	0.2	3

**Table 3:** The physical properties of modified asphalts before thermal oxidation aging.

Sample	Penetration (0.1 mm) (25 °C, 100 g, 5 s)	Softening Point (°C)	Ductility (cm) (5 cm/min, 10 °C)
Asphalt	63.2 ± 0.1	47.7 ± 0.3	62.0 ± 0.4
A-1PG	52.4 ± 0.3	50.9 ± 0.2	15.5 ± 0.6
A-2PG	47.0 ± 0.2	51.3 ± 0.1	15.7 ± 0.6
A-3PG	44.0 ± 0.3	52.6 ± 0.6	17.6 ± 0.8
A-4PG	40.2 ± 0.6	54.9 ± 0.3	18.8 ± 0.5
A-3PG	44.0 ± 0.1	52.6 ± 0.3	17.6 ± 0.9
A-3PG-0.05	44.1 ± 0.4	56.6 ± 0.1	16.5 ± 0.3
A-3PG-0.1	44.4 ± 0.1	57.6 ± 0.2	14.1 ± 0.6
A-3PG-0.2	45.4 ± 0.2	59.2 ± 0.7	13.1 ± 0.8

**Table 4:** The physical properties of modified asphalts after thermal oxidation aging.

Sample	Penetration (0.1 mm) (25 °C, 100 g, 5 s)	Softening Point (°C)	Ductility (cm) (5 cm/min, 10 °C)
Asphalt	41.4 ± 0.3	54.4 ± 0.2	25.3 ± 0.6
A-3PG	30.4 ± 0.7	58.8 ± 0.6	8.0 ± 0.9
A-3PG-0.05	30.5 ± 0.2	61.5 ± 0.3	7.7 ± 0.7
A-3PG-0.1	30.9 ± 0.7	61.9 ± 0.2	7.5 ± 0.3
A-3PG-0.2	32.0 ± 0.3	62.5 ± 0.7	7.2 ± 0.6

## Data Availability

Not applicable.

## References

[1] Liang B., Shi K., Niu Y.F., Liu Z.C., Zheng J.L. (2020). Probing the modification mechanism of and customized processing design for SBS-modified asphalts mediated by potentiometric titration. Constr. Build. Mater..

[2] Behnood A., Olek J. (2017). Rheological properties of asphalt binders modified with styrene-butadiene-styrene (SBS), ground tire rubber (GTR), or polyphosphoric acid (PPA). Constr. Build. Mater..

[3] Xiao F.P., Amirkhanian S., Wang H.N., Hao P.W. (2014). Rheological property investigations for polymer and polyphosphoric acid modified asphalt binders at high temperatures. Constr. Build. Mater..

[4] Bensason S., Stepanov E.V., Chum S.P., Hiltner A., Baer E. (1997). Deformation of elastomeric ethylene-octene copolymers. Macromolecules.

[5] Bensason S., Minick J., Moet A.A., Chum S.P., Hiltner A., Baer E. (1996). Classification of homogeneous ethylene-octene copolymers based on comonomer content. J. Polym. Sci. Polym. Phys..

[6] Behnood A., Gharehveran M.M. (2019). Morphology, rheology, and physical properties of polymer-modified asphalt binders. Eur. Polym. J..

[7] Li M.R., Chen X., Cong P.L., Luo C.J., Zhu L.Y., Li H.Y., Zhang Y.M., Chao M., Yan L.K. (2022). Facile synthesis of polyethylene-modified asphalt by chain end-functionalization. Compos. Commun..

[8] Polacco G., Stastna J., Biondi D., Zanzotto L. (2006). Relation between polymer architecture and nonlinear viscoelastic behavior of modified asphalts. Curr. Opin. Colloid Interface Sci..

[9] Behnood A., Olek J. (2017). Stress-dependent behavior and rutting resistance of modified asphalt binders: An MSCR approach. Constr. Build. Mater..

[10] Nien Y.H., Yeh P.H., Chen W.C., Liu W.T., Chen J.H. (2008). Investigation of flow properties of asphalt binders containing polymer modifiers. Polym. Compos..

[11] Padhan R.K., Leng Z., Sreeram A., Xu X. (2020). Compound modification of asphalt with styrene-butadiene-styrene and waste polyethylene terephthalate functionalized additives. J. Clean. Prod..

[12] Navarro F.J., Partal P., García-Morales M., Martínez-Boza F.J., Gallegos C. (2007). Bitumen modification with a low-molecular-weight reactive isocyanate-terminated polymer. Fuel.

[13] Research Institute of Highway Ministry of Transport (2011). Standard Test Methods of Bitumen and Bituminous Mixtures for Highway Engineering.

[14] Qu Y.D., Chen Y.H., Ling X.Y., Wu J.L., Hong J.T., Wang H.T., Li Y.J. (2022). Reactive micro-crosslinked elastomer for supertoughened polylactide. Macromolecules.

[15] Zhang Y.J., Liu X.Y., Li Y.Q., Wu D.F., Zhang M. (2023). Understanding the fracture toughness of gadolinium- and lead-containing plexiglass. Polym. Eng. Sci..

[16] Ouyang C.F., Gao Q., Shi Y.T., Shan X.Q. (2011). Compatibilizer in waste tire powder and low-density polyethylene blends and the blends modified asphalt. J. Appl. Polym. Sci..

[17] Padhan R.K., Sreeram A. (2018). Enhancement of storage stability and rheological properties of polyethylene (PE) modified asphalt using cross linking and reactive polymer based additives. Constr. Build. Mater..

[18] Yu C.H., Hu K., Yang Q.L., Wang D.D., Zhang W.G., Chen G.X., Kapyelata C. (2021). Analysis of the storage stability property of carbon nanotube/recycled polyethylene-modified asphalt using molecular dynamics simulations. Polymers.

[19] Li J., Zhang Y.X., Zhang Y.Z. (2008). The research of GMA-g-LDPE modified Qinhuangdao bitumen. Constr. Build. Mater..

[20] Redelius P., Soenen H. (2015). Relation between bitumen chemistry and performance. Fuel.

[21] Zhang Y.J., Wang C.H., Wu D.F., Guo X.T., Yu L., Zhang M. (2021). Probing the effect of straight chain fatty acids on the properties of lead-containing plexiglass. React. Chem. Eng..

[22] Ge D.D., Yan K.Z., You L.Y., Wang Z.X. (2017). Modification mechanism of asphalt modified with Sasobit and Polyphosphoric acid (PPA). Constr. Build. Mater..

[23] Zhang M.Y., Hao P.W., Dong S., Li Y., Yuan G.A. (2020). Asphalt binder micro-characterization and testing approaches: A review. Measurement.

[24] Li M.R., Luo C.J., Zhu L.Y., Li H.Y., Cong P.L., Feng Y.Y., Yan L.K. (2022). A novel epoxy-terminated polyethylene modified asphalt with low-viscosity and high storage stability. Constr. Build. Mater..

[25] Azarhoosh A., Koohmishi M. (2020). Investigation of the rutting potential of asphalt binder and mixture modified by styrene-ethylene/propylene-styrene nanocomposite. Constr. Build. Mater..

[26] Xiao Y., Chang X.W., Yan B.X., Zhang X.S., Yunusa M., Yu R.E., Chen Z.W. (2021). SBS morphology characteristics in asphalt binder and their relation with viscoelastic properties. Constr. Build. Mater..

[27] Gong Y.F., Pang Y.Z., Li F.Y., Jin W.D., Bi H.P., Ma Y.L. (2021). Analysis of the influence of SBS content and structure on the performance of SBS/CR composite modified asphalt. Adv. Mater. Sci. Eng..

[28] Lv S.T., Xia C.D., Yang Q., Guo S.C., You L.Y., Guo Y.P., Zheng J.L. (2020). Improvements on high-temperature stability, rheology, and stiffness of asphalt binder modified with waste crayfish shell powder. J. Clean. Prod..

[29] Vargas M.A., Vargas M.A., Sánchez-Sólis A., Manero O. (2013). Asphalt/polyethylene blends: Rheological properties, microstructure and viscosity modeling. Constr. Build. Mater..

[30] Sengoz B., Topal A., Isikyakar G. (2009). Morphology and image analysis of polymer modified bitumens. Constr. Build. Mater..

[31] Dong F.Q., Zhao W.Z., Zhang Y.Z., Wei J.M., Fan W.Y., Yu Y.J., Wang Z. (2014). Influence of SBS and asphalt on SBS dispersion and the performance of modified asphalt. Constr. Build. Mater..

[32] Yan C.Q., Huang W.D., Xiao F.P., Lv Q. (2017). Influence of polymer and sulphur dosages on attenuated total reflection Fourier transform infrared upon Styrene-Butadiene-Styrenemodified asphalt. Road Mater. Pavement Des..

[33] Luo Y.H., Li G.L., Chen L., Hong F.F. (2023). Preparation and evaluation of bacterial nanocellulose/hyaluronic acid composite artificial cornea for application of corneal transplantation. Biomacromolecules.

[34] Geng J.G., Meng H.H., Xia C.Y., Chen M.Y., Lu T.Y., Zhou H. (2021). Effect of dry-wet cycle aging on physical properties and chemical composition of SBS-modified asphalt binder. Mater. Struct..

[35] Peng C., Guo C., You Z.P., Xu F., Ma W.B., You L.Y., Li T.J., Zhou L.Z., Huang S.F., Ma H.C. (2020). The effect of waste engine oil and waste polyethylene on UV aging resistance of asphalt. Polymers.

[36] Li L.M., Guo Z.Y., Ran L.F., Zhang J.W. (2020). Study on low-temperature cracking performance of asphalt under heat and light together conditions. Materials.

[37] Wang S.F., Xie Y.G. (2016). Crumb tire rubber polyolefin elastomer modified asphalt with hot storage stability. Prog. Rubber Plast. Recycl. Technol..

[38] Liu S.J., Zhou S.B., Xu Y.S. (2018). Evaluation of cracking properties of SBS-modified binders containing organic montmorillonite. Constr. Build. Mater..

[39] Ouyang C.F., Wang S.F., Zhang Y., Zhang Y.X. (2006). Improving the aging resistance of styrene-butadienestyrene tri-block copolymer modified asphalt by addition of antioxidants. Polym. Degrad. Stab..

[40] Liu S., Shan L.Y., Li G.N., Underwood B.S., Qi C. (2022). Molecular-based asphalt oxidation reaction mechanism and aging resistance optimization strategies based on quantum chemistry. Mater. Des..

[41] Smith L.M., Aitken H.M., Coote M.L. (2018). The fate of the peroxyl radical in autoxidation: How does polymer degradation really occur?. Acc. Chem. Res..

[42] Li J.W., Ye X., Zhao Y.K., Yang D., Li D.D., Han C.C., Li X. (2022). Structure-performance evolution on thermal-oxidative aging of CeO_2_/SBS co-modified asphalt. Int. J. Pavement Eng..

[43] Pipintakos G., Soenen H., Ching H.Y.V., Velde C.V., Van Doorslaer S., Lemière F., Varveri A., Van den Bergh W. (2021). Exploring the oxidative mechanisms of bitumen after laboratory short- and long-term ageing. Constr. Build. Mater..

[44] Zhang Y.J., Chen Z.Y., Zhao R., Wang K., Wu D.F., Wang C.H., Zhang M. (2022). Insight into the role of free volume in irradiation resistance to discoloration of lead-containing plexiglass. J. Appl. Polym. Sci..

